# Survival in acute myeloid leukemia is associated with NKp44 splice variants

**DOI:** 10.18632/oncotarget.8782

**Published:** 2016-04-18

**Authors:** Avishai Shemesh, Michael Brusilovsky, Uzi Hadad, Omri Teltsh, Avishay Edri, Eitan Rubin, Kerry S. Campbell, Benyamin Rosental, Angel Porgador

**Affiliations:** ^1^ The Shraga Segal Department of Microbiology, Immunology and Genetics, Faculty of Health Sciences, Ben-Gurion University of the Negev, Beer Sheva, Israel; ^2^ National Institute for Biotechnology in the Negev, Ben-Gurion University of the Negev, Beer Sheva, Israel; ^3^ Immune Cell Development and Function Program, Fox Chase Cancer Center, Philadelphia, PA, USA; ^4^ Institute for Stem Cell Biology and Regenerative Medicine, Stanford University School of Medicine and the Hopkins Marine Station, Stanford, CA, USA

**Keywords:** AML, NKp44, splice variants, isoforms, natural killer cells

## Abstract

NKp44 is a receptor encoded by the NCR2 gene, which is expressed by cytokine-activated natural killer (NK) cells that are involved in anti-AML immunity. NKp44 has three splice variants corresponding to NKp44^ITIM+^ (NKp44-1) and NKp44^ITIM−^ (NKp44-2, and NKp44-3) isoforms. RNAseq data of AML patients revealed similar survival of NKp46^+^NKp44^+^ and NKp46^+^NKp44^−^ patients. However, if grouped according to the NKp44 splice variant profile, NKp44-1 expression was significantly associated with poor survival of AML patients. Moreover, activation of PBMC from healthy controls showed co-dominant expression of NKp44-1 and NKp44-3, while primary NK clones show more diverse NKp44 splice variant profiles. Cultured primary NK cells resulted in NKp44-1 dominance and impaired function associated with PCNA over-expression by target cells. This impaired functional phenotype could be rescued by blocking of NKp44 receptor. Human NK cell lines revealed co-dominant expression of NKp44-1 and NKp44-3 and showed a functional phenotype that was not inhibited by PCNA over-expression. Furthermore, transfection-based overexpression of NKp44-1, but not NKp44-2/NKp44-3, reversed the endogenous resistance of NK-92 cells to PCNA-mediated inhibition, and resulted in poor formation of stable lytic immune synapses. This research contributes to the understanding of AML prognosis by shedding new light on the functional implications of differential splicing of NKp44.

## INTRODUCTION

Acute myeloid leukemia (AML) is the most common acute leukemia in American adults. AML quickly progresses and can result in death within a few months if left untreated. [1, 2], [3], [4] One of the major cell populations involved in anti-AML immune responses is CD3^−^CD56^+^ innate lymphoid NK cells. [5–11] It has been previously shown that the status of NK cells in AML is critical for patients’ survival and that AML leukemic cells can induce impaired NK cell function. [12–14] NK cell function is regulated through a delicate balance of activation vs. inhibitory signals that is sustained by an array of cell surface receptors engaging ligands on target cells. [15] While activation receptors (e.g. NKG2D and NCRs) mostly recognize stress and viral induced ligands, inhibitory receptors (KIRs/LIRs/NKG2A) interact with class I HLA molecules and transmit an inhibitory signal via immunoreceptor tyrosine-based inhibitory motifs (ITIMs). [16–18]

NKp44 (CD336) is a cell surface glycoprotein with one IgG type V-like extracellular domain, is encoded by the NCR2 gene located on chromosome 6, and has no homolog in the mouse genome. [19–21] NKp44/NCR2 belongs to a family of receptors called Natural Cytotoxicity Receptors (NCRs), which also includes NKp30/NCR3 and NKp46/NCR1. However, while NKp46 and NKp30 are constitutively expressed on NK cells, NKp44 is not expressed on resting peripheral NK cells, but can be up-regulated upon NK cell activation by IL-2 or IL-15. [22, 23] Among NKp44 ligands are heparan sulfate (HS) and various viral proteins. [24–27] Recently, two new intracellular proteins were identified as ligands for NKp44; a specific MLL5 isoform and PCNA. [28, 29] The specific MLL5 isoform leads to NK cell activation. PCNA over-expression is abundant in cancers and is directly associated with cancer virulence. Surprisingly, PCNA expression was found to inhibit NK cell function upon engagement with NKp44, potentially promoting cancer escape from immune detection. [29–31] The PCNA-mediated inhibitory effect was shown to require the ITIM motif that can be transcribed on the cytoplasmic tail of the NKp44 receptor, but not the receptor-associated adaptor protein DAP12, which mediates NKp44 activation signaling. [29, 32], [33]

Alternative splicing allows a single gene to be translated into multiple forms of the same protein (isoforms) and therefore constitutes a regulatory mechanism that leads to diverse function of proteins encoded by the same gene. [34] Until now, a few receptors expressed on NK cells were found to be regulated by alternative splicing. [35, 36] Human NKG2D can be expressed as a truncated receptor lacking the ligand-binding ecto-domain, thereby competing with the full-length receptor to disrupt its function. [37] In addition, predominant expression of a NKp30 splice variant, designated NKp30c, was shown to indicate poor prognosis of patients with gastrointestinal stromal tumors. [38] The effect was linked to production of the immune inhibitory cytokine, IL-10, by NKp30c-expressing NK cells. Alternative splicing of NKp44 mRNA results in three splice variants, which can be distinguished by the presence or absence of an ITIM in the cytoplasmic portion of the receptor; NKp44^ITIM+^ (NKp44-1) and NKp44^ITIM−^ (NKp44-2&3) isoforms. [32] While NKp44-1 has been associated with the inhibitory effect of PCNA, inhibition was never tested for the other two NKp44 splice variants, NKp44-2 and NKp44-3, which lack the ITIM motif. While these alternatively spliced forms of NCRs have been recognized, they have been minimally characterized.

Here, we demonstrate that the expression of ITIM^+^ alternative splice variants of the NCR2 gene, NKp44 are associated with poor survival of AML patients. Furthermore, we show that the balance between NKp44^ITIM+^ and NKp44^ITIM−^ splice variants can lead to diverse function of NK cells and influence the outcome of NK cell-target cell interactions.

## RESULTS

### Poor survival of AML patients with a NKp44-1 splice variant profile

**Figure 1:**
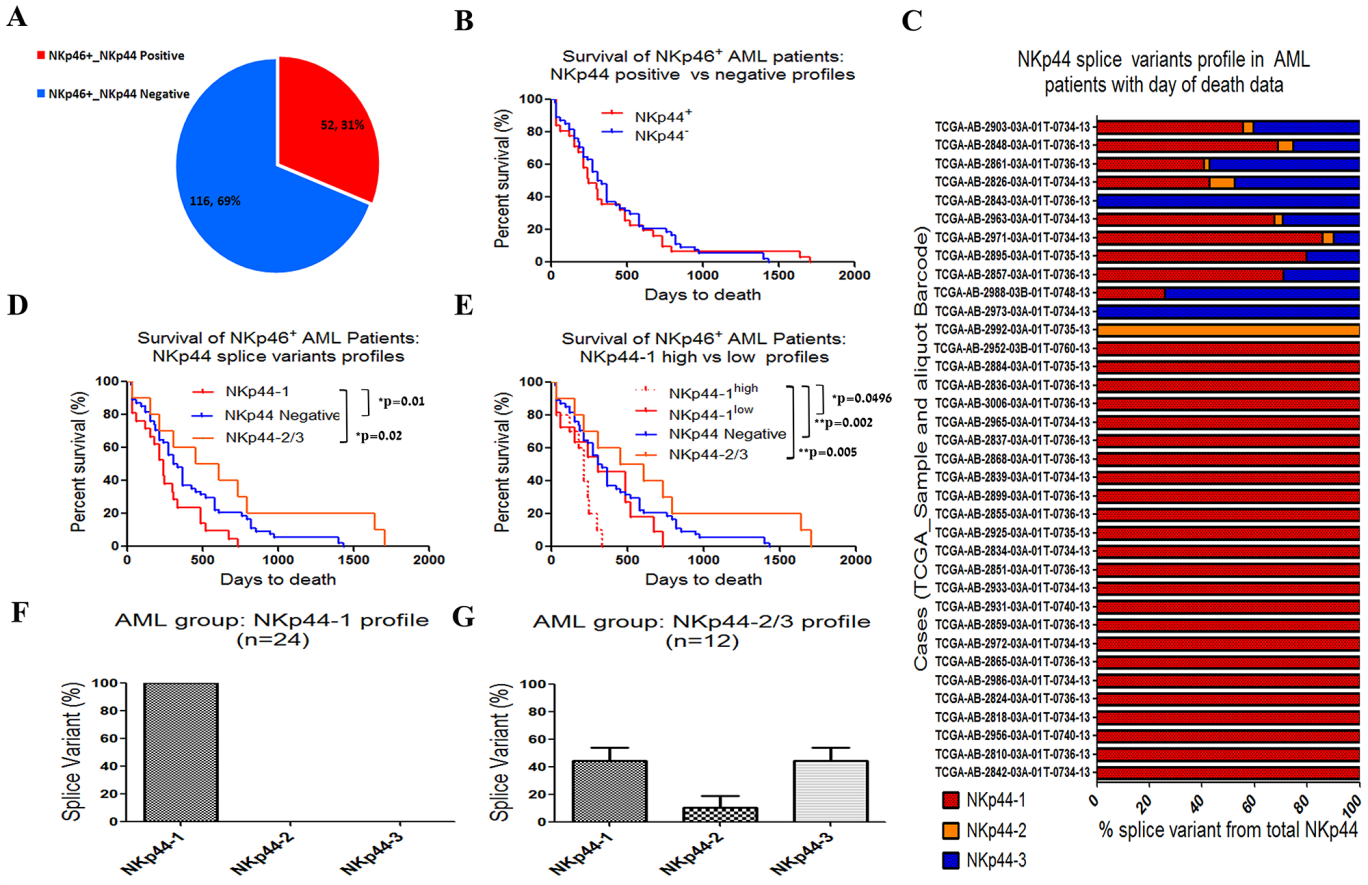
Poor survival of AML patients with the NKp44-1 profile RNAseq analysis of PB samples obtained from AML patients (TCGA data): **A.** Proportions of NKp46^+^NKp44^+^ (n=51) and NKp46^+^NKp44^−^ (n=115) patients from all NKp46^+^ AML cases. **B.** Of the AML patients in panel (A), only 36 patients from the NKp46^+^NKp44^+^ group (Red) and 60 patients from the NKp46^+^NKp44^−^ group (Blue) had the “day to death” information, which were plotted (difference is not statistically significant). **C.** Percentage of NKp44 splice variants [NKp44-1 (Red), NKp44-2 (Orange), NKp44-3 (Blue)] from the total NKp44 mRNA expression for NKp46^+^NKp44^+^ AML cases that included the day to death information (n=36). Each line designates the analysis for one patient TCGA sample and corresponding barcode. **D.** Survival of NKp46^+^ AML cases with a profile of NKp44-1 (red, n=24) vs. NKp44-2/3 (orange, n=12) vs. NKp44^−^ (blue, n=60). **E.** Survival of NKp46^+^ AML cases with differential profile of NKp44-1 after normalization to NKp46/NCR1: NKp44-1^high^(dotted red, n=12) vs. NKp44-1^low^ (solid red, n=12). NKp44-2/3 (green, n=12) vs. NKp44^−^ (blue, n=60) profiles (from E) are re-plotted for the ease of comparison. **F.** Incidence of NKp44 splice variants in the NKp44-1profile group (F) and the NKp44-2/3 profile group **G.** The NKp44-1 profile group shows solitary mRNA expression of the NKp44-1 splice variant while the NKp44-2/3 profile group shows similar mRNA levels of NKp44-1 and NKp44-3 splice variants. A highly significant correlation was shown between the NCR2 mRNA expression and the total NKp44 expression (Pearson r = 0.9996, p < 0.0001, n = 36, graph not shown). Percent survival vs “day of death” statistics were calculated using the Log-rank (Mantel-Cox) Test. mRNA expression statistics were performed using Unpaired t test, two-tail.

We and others had previously shown that PCNA expression can be utilized by cancer cells to suppress NK cell activity, through interaction with the NK receptor NCR2/NKp44. [29, 31, 39] To further explore this finding, we performed a retrospective analysis on RNAseq data obtained from peripheral blood (PB) samples of AML patients deposited in the TCGA database. RNAseq data from 173 PB samples was filtered for the presence of NK cells using expression of the NK cell-associated receptor, NKp46 (*i.e.* total NKp46). The 164 of 173 cases positive for NKp46 were chosen for further analysis, and 31% of these were NKp44^+^ (*i.e.* total NKp44/NCR2; Figure 1A). We then tested the contribution of NKp44 expression to the survival of AML patients by comparing NKp46^+^NKp44^+^ to NKp46^+^NKp44^−^ groups. From all NKp46^+^ AML cases, only 60 cases of NKp46^+^NKp44^−^ and 36 cases of NKp46^+^NKp44^+^ had the “days to death” data deposited in the TCGA. No difference, however, was seen in the percent survival between the NKp46^+^NKp44^+^ and NKp46^+^NKp44^−^ cases groups (Figure 1B). To further investigate the role of NKp44 in AML associated morbidity, we looked into the expression of NKp44 splice variants, since NCR2 mRNA can be spliced into three different splice variants: NKp44-1, NKp44-2 and NKp44-3. [32]

The percentage of RNAseq-based expression of each of the NKp44 splice variants from total NKp44 mRNA expression in the NKp46^+^NKp44^+^ group was calculated for each patient (=line) with “day to death” data, as detailed in Figure 1C. We observed that individual AML patients manifested a broad spectrum of NKp44 splice variant expression profiles, ranging from expression of a single NKp44 splice variant to expression of a mix of splice variants. Two thirds of the NKp46^+^NKp44^+^cases with “day to death” data expressed only the NKp44-1 splice variant (Figure 1C). Thus, we defined the NKp46^+^ NKp44-1^+^-only samples as having a NKp44-1 profile, whereas the NKp44-2/3 profile was defined to include all other NKp46^+^ samples expressing NKp44-2 and/or NKp44-3 (with or without expression of NKp44-1). NKp46^+^NKp44^−^ samples were defined as a NKp44^−^ profile. Figure 1D shows that survival of the NKp44-1 profile group was significantly lower than the NKp44^−^ and the NKp44-2/3 profile groups. To better characterize the association between the NKp44-1 expression levels and survival of AML patients, we further divided the NKp44-1 group by sorting in accordance with the expression levels of NKp44-1 (normalized to the expression of NCR1) by equally dividing into NKp44-1^high^ (top half, n=12) and NKp44-1^low^ (bottom half, n=12) subgroups. We then plotted percent survival of AML cases for NKp44-1^high^, NKp44-1^low^, NKp44-2/3 and NKp44^−^ profiles. The percent survival of NKp44-1^low^, NKp44-2/3 and NKp44^−^ profiles did not differ significantly. However, the patient group bearing the NKp44-1^high^ profile manifested a lower survival rate, which differed significantly from each of the other three groups (Figure 1E). We then checked if this lower survival rate could be a result of lower total NKp44/NCR2 expression. As expected, total NKp44/NCR2 gene expression was significantly higher in the NKp44-1^high^ group, as compared to the NKp44-1^low^ group (Supplementary Figure S1A.i). Yet, poor survival of the NKp44-1^high^ group could not be attributed to total NKp44 expression since the NKp44-2/3 profile group showed a significantly higher expression of total NKp44, as compared to the NKp44-1^high^ profile, mostly due to a significant contribution of NKp44-2 and NKp44-3 splice variants. Moreover, NKp44 total mRNA expression can be subject to alterations in the number of cells, hence we focused on the NKp44 splice variants profile of each patient.

We further considered that poor survival of the NKp44-1 profile group could be a consequence of parameters other than the NKp44 splice variants profile. To examine this further, we investigated contributions of other NCRs (NKp46 and NKp30 and related splice variants, NKp30a, NKp30b, and NKp30c). mRNA expression data obtained from the TCGA database were analyzed, and the NKp44-1 profile group, NKp44-2/3 profile group and NKp44 negative profile group were compared (NKp46^+^ cases with “day to death” data), but no significant differences were detected (Supplementary Figure S1A, S1B). We then expanded our analysis to known AML risk factors and AML cytogenetic risk groups [4], but no significant differences were observed to explain the significant poor survival observed for the NKp44-1 profile (Supplementary Table S1). Clearly, we cannot rule out other factors that may affect survival in AML. Furthermore, it must be considered that the TCGA database is comprised of newly diagnosed AML cases that subsequently experienced diverse treatment regimens, and subsequent therapies may have significantly influenced outcomes. Nonetheless, our analysis suggests that the NKp44 splice variants profile may serve as an early prognostic factor for AML.

We next plotted the patient groups according to the incidence of individual NKp44 splice variants, which demonstrated two distinct NKp44 splice variant expression profiles. As shown in Figure 1F and 1G, the NKp44-1 profile group exhibits solitary expression of NKp44-1, while the NKp44-2/3 profile group exhibits co-dominant expression of NKp44-1 and NKp44-3.

Note that the NKp44 receptor is typically expressed by NK cells, yet other cells were also reported to express NKp44, including pDC and non-NK innate lymphoid cells (ILC). [40, 41] Therefore, we compared the correlation of NKp44 gene expression with expression of genes associated with the cells reported to express NKp44. Expression of the NK cell marker CD56 and NKp44 were significantly correlated, while expression of genes characterizing DC were not correlated with the NKp44 expression. We then checked the correlation of NKp44/NCR2 to NKp46/NCR1, but we did not show a significant correlation, which may reflect the nature of NKp44 as an inducible receptor (data not shown). However, when we tested the correlation of NKp46/NCR2 to CD56 and other DC markers, NKp46 was highly correlated to CD56 but not to known DC markers (Supplementary Figure S2). Hence, these data indicate that NKp44 mRNA expression in AML is associated with NK cells rather than DC. While we cannot rule out the possible involvement of other ILC, they are rare in peripheral blood [41]

### Human pNK cells and clones exhibit diverse NKp44 splice variant profiles

**Figure 2:**
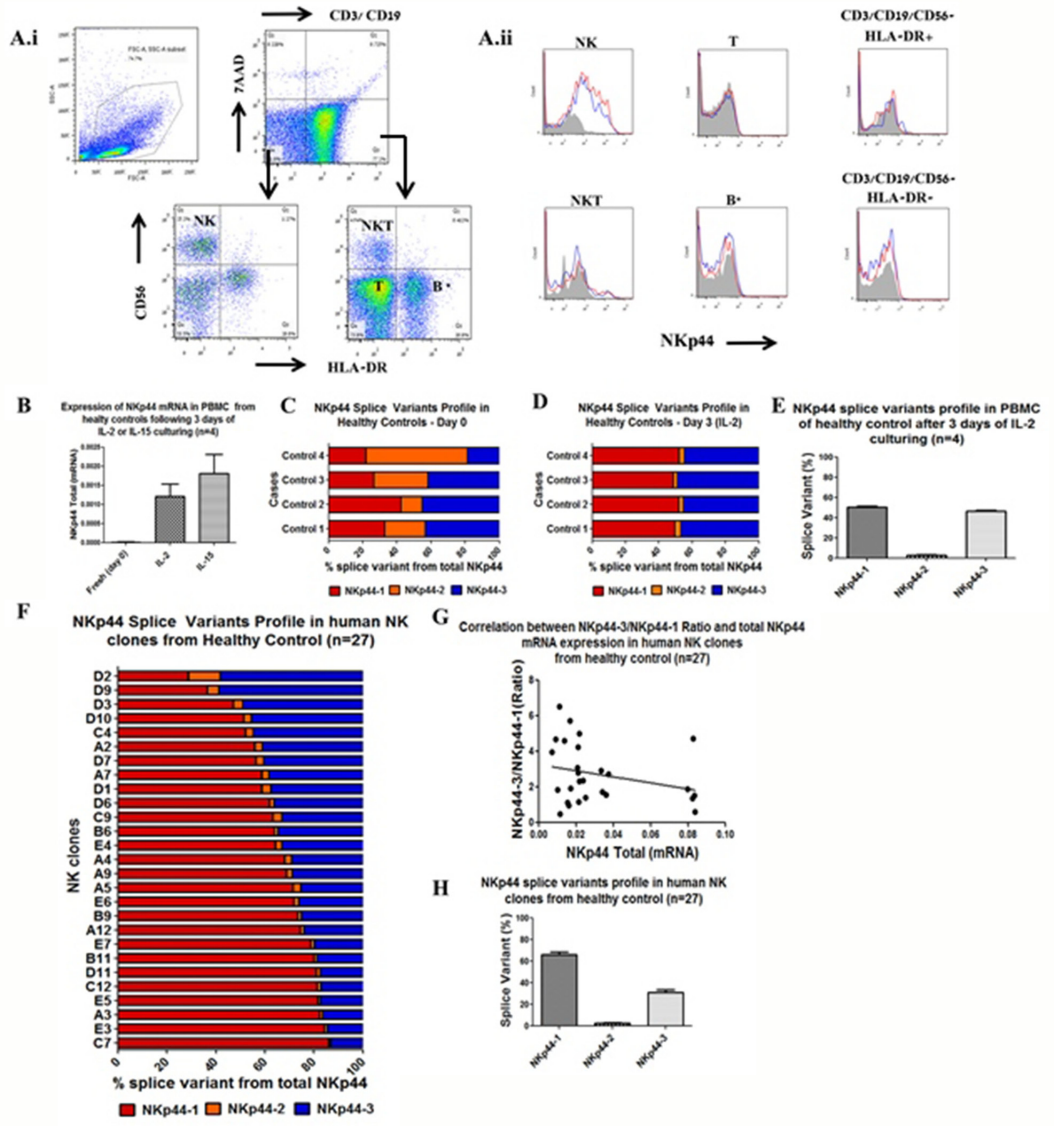
Human primary NK cells and clones exhibit diverse NKp44 splice variant profiles **A.i.** Flow cytometry-based analysis of PBMC, representative histogram from a healthy donor (n=4). Gating of PBMC subpopulations was performed using the following markers: 7AAD, CD3, CD19, CD56, HLA-DR and NKp44. **A.ii.** Total NKp44 protein expression is shown by histogram of each PBMC subpopulation on day 0 (filled grey) and following 3 days of culture with IL-2 (blue line) or with IL-15 (red line). * Some CD4 T cells express HLA-DR. **B.** qPCR analysis of total NKp44 mRNA expression on day 0 PBMC and following 3 days of culture with IL-2 or IL-15. qPCR analysis of NKp44 splice variants percentages in PBMC: **C.** day 0, **D.** after 3 days of IL-2 culturing. NKp44-1 (red), NKp44-2 (orange), NKp44-3 (blue). **E.** NKp44 splice variant profile after IL-2 culturing, of the 4 healthy controls pooled together shows co-dominant expression of NKp44-1 and NKp44-3. **F.** NKp44 splice variants profiles of 27 human NK clones from one healthy control. Human NK cell clones were cultured in the presence of IL-2, PHA and irradiated PBMC and RPMI8866 cells. NKp44-1 (red), NKp44-2 (orange), NKp44-3 (blue). **G.** Lack of a correlation between NKp44 total mRNA expression and NKp44-3/NKp44-1 ratio in NK cells clones (Pearson r = 0.09, p =0.6985, n=27). **H.** NKp44 splice variant profiles of the 27 human NK cell clones of one healthy control pooled together shows dominant expression of NKp44-1 relative to NKp44-2 and NKp44-3.

Human NK cells in peripheral blood do not readily express surface NKp44 at the protein level, yet, following activation with IL-2 or IL-15, cell surface NKp44 protein is expressed. [42] Therefore, we purified PBMCs from healthy donors and cultured them in the presence of IL-2/IL-15 to induce NKp44 protein expression. As expected, fresh PBMCs were not stained with specific mAb to NKp44, while PBMCs cultured for 3 days with either IL-2/IL-15 manifested positive staining; NKp44 expression was found to be restricted to NK cells and a small fraction of NKT cells (Figure 2A). To assess and compare the expression of the different NKp44 splice variants in the fresh and 3-days-activated PBMCs, we employed qPCR analysis to assess total NKp44 mRNA and each of the NKp44 splice variants in four healthy control subjects. Indeed, NKp44 total mRNA levels were significantly increased after IL-2/IL-15 culture for 3 days (Figure 2B); qPCR analysis of NKp44 splice variants in freshly isolated PBMCs (day 0) showed low basal mRNA expression and similar ratios of the 3 NKp44 splice variants (Figure 2C). When we examined expression profiles of NKp44 splice variants in PBMCs following 3 days of culture with IL-2and IL-15 (data not shown for IL-15), NKp44-1 and NKp44-3 expression was similarly up-regulated, while levels of the NKp44-2 splice variant remained relatively low (Figure 2D, 2E). These results were consistent with the PBMC samples taken from all four healthy donors.

Since NK cells are a heterogeneous immune population, we investigated whether the IL-2/IL-15-mediated co-expression of NKp44-1 and NKp44-3 splice variants stems from co-expression of both mRNAs in individual NK cells or from co-existence of distinct NK cells clones expressing only NKp44-1 or NKp44-3. To answer this question, we purified NK cells from PBMCs and performed a single-cell sub-culture in the presence of IL-2, PHA and irradiated allogeneic feeder PBMCs. All of the 27 pNK clones analyzed were found to express both NKp44-1 and NKp44-3, although at different ratios, as shown in Figure 2F. Most of the clones showed dominance of NKp44-1 while only two clones predominantly expressed NKp44-3 (Figure 2F).

We then asked whether the up-regulation of total NKp44 can be linked to any particular ratio of NKp44 splice variants, by testing for a correlation between the total expression of NKp44 and the NKp44-3/NKp44-1 ratio. The observed wide range of ratios between NKp44-1 and NKp44-3 splice variants was not correlated (Pearson r = 0.09, p =0.6985, n=27) with the total expression of NKp44 (Figure 2G). Plotting of the pooled data of splice variant expression profiles from all clones revealed higher relative NKp44-1 expression in fresh PBMC samples, as compared to IL-2 or IL-15 activated PBMCs (Figure 2E, 2H).

### Dominant expression of NKp44-1 is associated with impaired functional phenotype of peripheral NK cells

**Figure 3:**
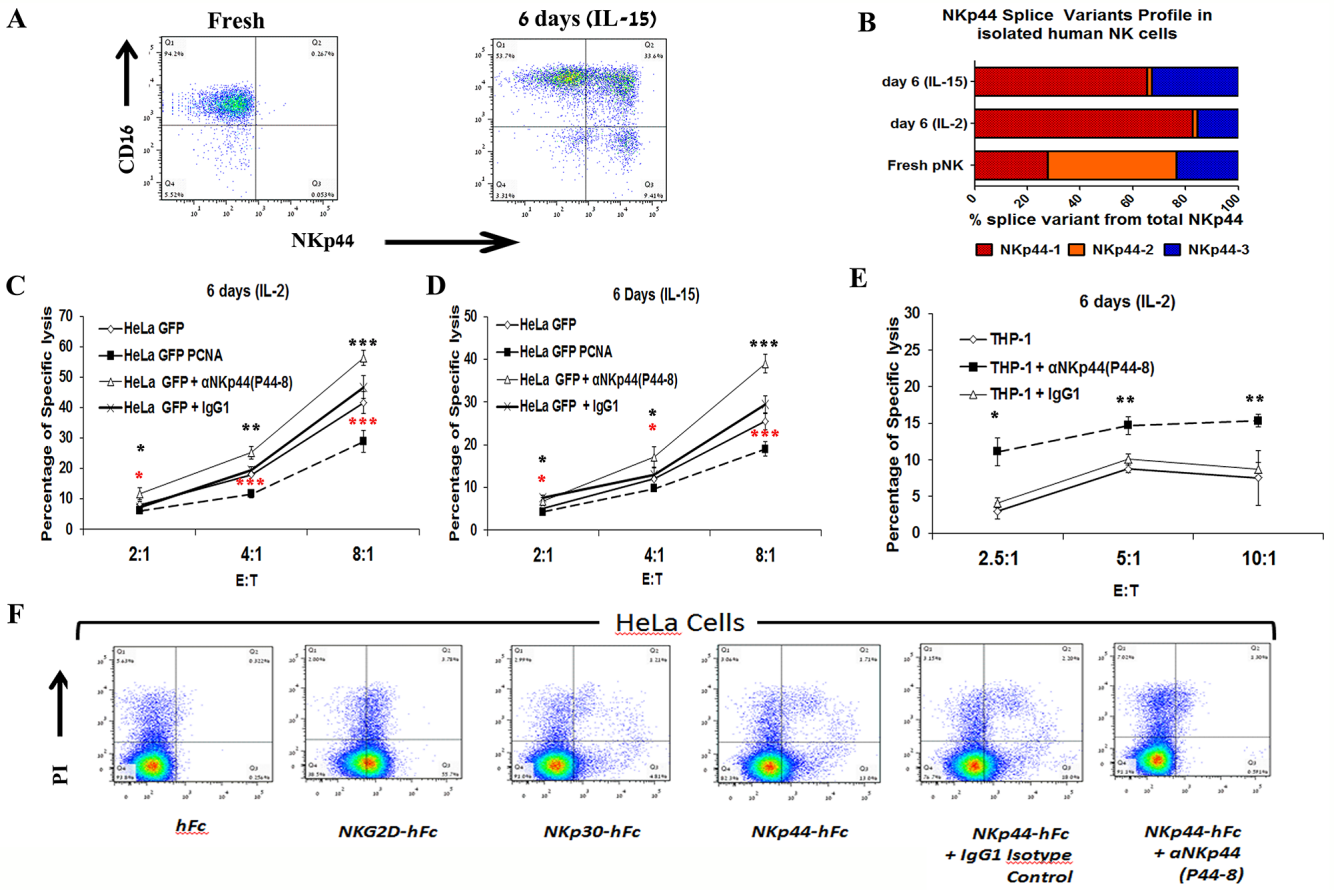
Dominant expression of NKp44-1 is associated with impaired functional phenotype of peripheral NK cells Primary human NK cells were isolated from blood and cultured for 6 days in the presence of IL-2 or IL-15. **A.** Flow-cytometry based analysis of CD3^−^/CD56^+^ NK cells for the markers CD16 and NKp44 on day 0 and after 6 days of culture with IL-15 (dot-plot for IL-2 is not shown). **B.** NKp44 splice variants profiles analyzed by qPCR in pNK cells at day 0 and following 6 days of treatment with IL-2 or with IL-15. NKp44-1 (Red), NKp44-2 (Orange), NKp44-3 (Blue). Function of 6 days IL-2 **C.**- or IL-15 **D.**-cultured primary human NK cells. Lysis of HeLa GFP PCNA cells was compared to lysis of HeLa GFP cells with or w/o blocking with anti-NKp44 mAb (p44-8). **E.** Lysis of the THP-1 AML cell line by 6 days IL-2-cultured primary human NK cells in the presence or absence of blocking anti-NKp44 mAb (p44-8). **F.** HeLa cells were stained with PI in order to discriminate between live and dead cells. Dot plots show the binding of NKG2D-Fc, NKp30-Fc and NKp44-Fc in the presence of anti-NKp44 blocking mAb (p44-8) or isotype control Ab. Relative to NKG2D-Fc binding, NKp30-Fc and NKp44-Fc show higher binding to dead cells as both have known intracellular ligands. P44-8 mAb but not the isotype control Ab, blocks the binding of NKp44-Fc to live and dead HeLa cells. hFc was use as a control for non-specific binding. Bars, ±SD. *, p<0.05; **, p<0.01; ***, p<0.001; Unpaired t-test, two-tail. The percentage of specific cytotoxicity was calculated using the formula: (experimental killing - spontaneous killing)/ (maximum killing - spontaneous killing) x100.

Following the profiling of NKp44 splice variants in human PBMC and NK clones from healthy controls, we isolated primary NK cells from PB in order to investigate the functional properties of NK cell clones with distinct NKp44 splice variant profiles. To test this, we cultured the isolated NK cells in the presence of IL-2 or IL-15 for 6 days. Indeed, NKp44 protein was not detected on the surface of resting CD3^−^/CD56^+^/CD16^+^ and CD3^−^/CD56^+^/CD16^−^ pNK cells by flow cytometry, but was up-regulated upon culture with IL-15 (Figure 3A) or IL-2 (data not shown). qPCR analysis of total NKp44 mRNA revealed cytokine-induced up-regulation, which was consistent with the induced expression of NKp44 surface protein, and mRNA for all NKp44 splice variants was increased (Supplementary Figure S3A, S3B, S3C, S3D). Analysis of the profiles of NKp44 splice variants in pNK cells that had been cultured with IL-2 and IL-15 revealed that the expression of NKp44-1 and NKp44-3 mRNAs as a fraction of total NKp44 mRNA were increased, while the expression of NKp44-2 was relatively low (Figure 3B). However, the NKp44-1 splice variant was predominantly expressed in the cultured NK cells, as compared with NKp44-3 (low) and NKp44-2 (dull) (Figure 3B). We have previously shown that IL-2-cultured pNK cells up-regulate surface expression of NKp44 protein, which leads to functional inhibition upon interaction with PCNA expressed by target cells. [29] Here, we show that IL-2 cultured pNK cells with a NKp44-1 dominant splice variant profile exhibited lower lytic activity towards PCNA-over-expressing HeLa target cells as compared to control HeLa cells (Figure 3C, HeLa GFP vs. HeLa GFP PCNA). The same functional outcome was observed also for IL-15 cultured pNK cells (Figure 3D, HeLa GFP vs. HeLa GFP PCNA). Both phenomena correlate with the dominance of NKp44-1 splice variant expression after culturing pNK with IL-2/IL-15 (Figure 3B). We next examined the effect of blocking NKp44 with P44-8 mAb and the resulting function of primary human NK cells cultured in IL-2/IL-15. Blocking of NKp44 resulted in a higher percent of target cell lysis, as demonstrated in direct cytolytic assays (Figure 3C, 3D).

To extend our findings to myeloid target cell line, we tested the NK-resistant THP-1 target AML-type cell line. The low lysis of THP-1 by primary human NK cells that had been cultured 6 days in IL-2 was significantly enhanced following incubation with anti-NKp44 mAb (Figure 3E). In contrast, NK-sensitive K562 CML-type cell line was killed very efficiently by the same NK cells (>80%) and thus incubation with the anti-NKp44 mAb did not show enhancement or reduction in lysis (data not shown). The differences between the sensitivity of THP-1 AML cell line and K562 CML cell line could be due to differences in MHC class-I expression (Supplementary Figure S3). [43]

The only known inhibitory ligand of NKp44 is PCNA, which is mostly intracellular. A soluble form of the receptor (NKp44-hFc) was found to bind readily to the surface of live cells and manifested enhanced binding to dead HeLa cells (Figure 3F). This pattern of enhanced staining of dead cells, compared to live cells, was also observed following staining with a soluble form of NKp30 (NKp30-hFc), which similarly to NKp44 has intracellular ligands. In accordance, the enhanced staining of dead cells was not observed for staining with soluble NKG2D (NKG2D-hFc), which does not have intracellular ligands (Figure 3F). The binding of NKp44-hFc to live and dead HeLa cells was blocked with anti-NKp44 mAb (p44-8) (Figure 3F), which is consistent with the capacity of this mAb to enhance the function of NK cells with a NKp44-1-dominant splice variant profile by blocking interaction with membrane-associated PCNA (Figures 3C, 3D, 3E).

Taken together, our findings indicate that the impaired functional phenotype of pNK cells that results in suppressed lysis of cancer target cells is associated with NKp44-1 dominant expression. Furthermore, we previously showed (Figure 1) that the solitary expression of the NKp44-1 splice variant in PB of AML patients was associated with reduced survival, which could indicate that excess expression of the NKp44-1 isoform is associated with functionally-deficient NK cells.

### Co-dominant expression of NKp44-1 and NKp44-3 leads to “resilient” NK function toward PCNA-expressing tumor cells

We have previously demonstrated that target cell cytotoxicity by the NK-92 cell line was not influenced by PCNA over-expression by target cells. [29] Therefore, we tested whether the NKp44 splice variant expression profile of NK-92 cells could explain this phenomenon and also examined the KHYG-1 NK cell line, which expresses higher levels of endogenous NKp44.

**Figure 4:**
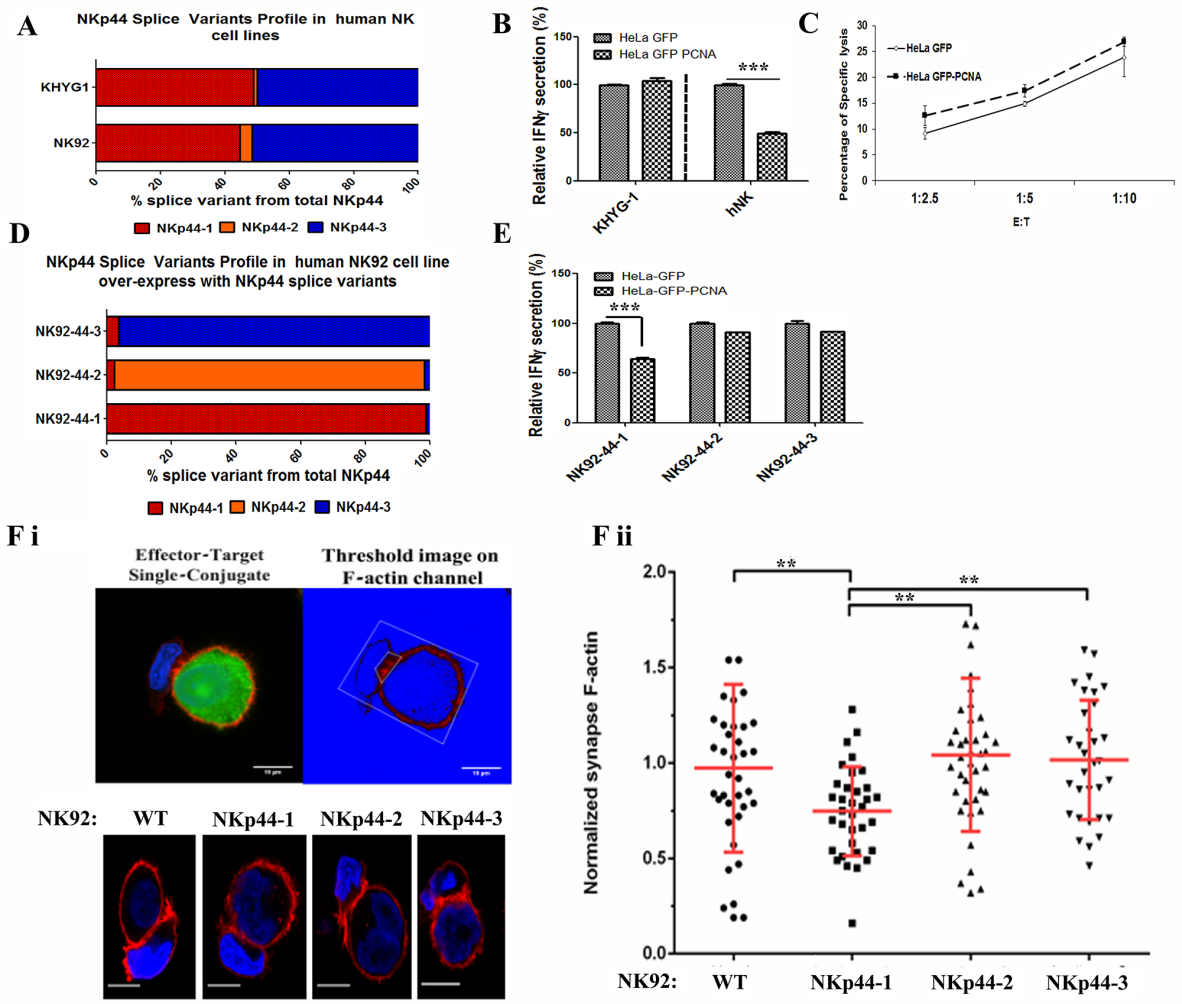
Co-dominant expression of NKp44-1 and NKp44-3 leads to “resilient” NK function toward PCNA-expressing tumor cells Human NK cells lines were tested for the percentage of NKp44 splice variants (qPCR analysis): **A.** NK-92 and KHYG-1, NKp44-1 (red), NKp44-2 (orange), NKp44-3 (blue). **B.** Function of KHYG-1 and 6-days IL-2 cultured primary human NK cells from healthy control. Relative IFNγ production by KHYG-1 and 6d IL-2 cultured hNK cells following 18h incubation with HeLa GFP or HeLa GFP PCNA target cells. **C.** Lysis assay of HeLa GFP and HeLa GFP PCNA target cells by KHYG-1 cells. **D.** qPCR analysis of NKp44 splice variants percentage in: NKp44 splice variant 1-transfected NK-92 cells (NK92-44-1), NKp44 splice variant 2-transfected NK-92 cells (NK92-44-2), and NKp44 splice variant 3-transfected NK-92 cells (NK92-44-3) cell lines [NKp44-1 (Red), NKp44-2 (Orange), NKp44-3 (Blue)]. **E.** IFNγ secretion by NK92-44-1, NK92-44-2, NK92-44-3 cell lines following 18h incubation with target cells. Results are normalized/relative to HeLa GFP (100%). **F.** Relative quantification of immune synapse specific F-actin accumulation. WT-NK-92, NK92-44-1,-2, and -3 cell lines were co-incubated on confocal chamber slides with CFSE-labeled HeLa target cells (green), fixed and permeabilized, and stained with Phalloidin. In representative images of NK-target interactions shown in panel F.i, pseudo color (black to white) was applied to indicate Phalloidin fluorescence intensity. Upper panel shows conjugation with relatively high level of Immune Synapse F-actin accumulation and lower panel shows low level of F-actin at the immune synapse. Images with over-saturated pixels were excluded from analysis. For image analysis, background fluorescence noise was eliminated using ImageJ mean threshold algorithm (indicated by blue background). In order to neglect fluorescent signal originated from target cell F-actin and variation in staining intensity; gated synapse F-actin MFI was divided by total conjugation MFI F.ii). Bars, ±SD. Unpaired t-test, two-tail. Images were acquired using a FluoView 1000 (Olympus) laser scanning confocal microscope equipped with an UPLSAPO 60X O NA:1.35 objective lens. Image analysis was done using Fiji software. [59]

We first tested the expression of NKp44 splice variants by NK-92 and KHYG-1 (Figure 4A). Both cell lines showed a similar profile, which was characterized by co-dominant expression of NKp44-1 and NKp44-3 splice variants with dull expression of NKp44-2 splice variant. This NKp44 splice variant profile of NK-92 cells could explain the resilient response toward PCNA over-expressing target cells. [29] Consistent with the results of NK-92 cells, IFNγ secretion by KHYG-1 cells was not inhibited by PCNA-overexpressing HeLa cells (Figure 4B). Similarly, PCNA over-expression by target cells did not inhibit lysis by KHYG-1 cells (Figure 4C), as compared to lysis by IL-2 cultured pNK (Figure 3C). These results are in sharp contrast to IL-2-cultured pNK cells that manifest dominant expression of NKp44-1, and in accordance, exhibit reduced IFNγ secretion when stimulated by PCNA-over-expressing HeLa cells (Figure 4B). To further assess the effect of each single NKp44 splice variant, we transfected NK-92 cells with cDNA encoding for each of the three NKp44 splice variants. As expected, the resulting NKp44 splice variant profile of the three NK-92 transfectants, (i.e. NK92-44-1, NK92-44-2 and NK92-44-3) showed a dominant expression of the transfected splice variant (Figure 4D). When tested in the functional assay, IFNγ secretion by NK92-44-1 cells, but not NK92-44-2/44-3 cells, was inhibited by over-expression of PCNA by target cells (Figure 4E).

We next explored whether target PCNA-impaired and target PCNA-resilient NK cell functional phenotypes associated with altered NKp44 splice variant profiles can influence the cytoskeleton rearrangement that is necessary for the formation of stable lytic immune synapses. We examined stable lytic immune synapse formation in each of the NKp44 splice variant-transfected NK-92 cell lines. NK-92 WT (parental) or, NK92-44-1/44-2/44-3 effectors were co-incubated with HeLa cells expressing CFP alone or CFP-PCNA fusion protein, and the relative accumulation of F-actin at the immune synapse was assessed in NK-92 cells over-expressing each splice variant (Figure 4Fi). We found that over-expression of the NKp44-1 splice variant resulted in a significant reduction (−22.6%±3.9%) in the relative amount of F-actin accumulating at the immune synapse in interactions of NK92-44-1 cells with PCNA over-expressing target cells (Figure 4Fii). In contrast, NK-92 cells transfected with splice variants 2 or 3 showed no significant decrease in F-actin accumulation (+6.9% and +4.6%, respectively) as compared to the control NK-92 WT effector cells (Figure 4Fii). Therefore, only the NK-92 cells predominantly expressing the NKp44-1 splice variant exhibited suppressed F-actin accumulation at the immune synapse with PCNA expressing target cells.

## DISCUSSION

NK cells are involved in anti-AML immune responses and functional impairment of NK cells can affect the clinical outcome of AML patients. [12-14, 44] The NKp44 receptor, along with the NKp46 and NKp30 receptors, belongs to the NCRs, which are predominantly expressed in innate lymphoid cells and are important activating receptors on pNK cells. [19, 22] In previous studies of NKp46 and NKp30 expression in AML patients, down-regulation of their expression was linked to impaired NK cell function and poor prognosis, while up-regulation was associated with enhanced NK cell function and better prognosis of the AML patients. [14, 44] In contrast, although NKp44 expression was studied on NK cells from AML patients, a possible link between AML immunity and NKp44 expression by NK cells was not previously reported. [14] The current study analyzed NKp44 expression and AML prognosis. Our initial results showed no correlation between NKp44 expression in PB samples and AML outcome. Thus, we could conclude that NKp44 expression in the blood of AML patients has no contribution to prognosis. However, when we deepened our studies into NKp44 splice variant profiles we realized that the story is more complex. Specifically, we showed that poor prognosis of AML patients is associated with high expression of NKp44-splice variant 1 in the patient blood. We connected these results to the presence of a cytoplasmic ITIM in the NKp44-1 isoform, as compared to NKp44-2&3 isoforms. We further showed that the presence of NKp44-1 isoform significantly suppresses F-actin aggregation during the formation of a stable NK cell immunological synapse with PCNA-expressing target cells. [13] We then associated the NK cell functional inhibition conferred by expression of the NKp44-1 isoform to PCNA expression by target cancer cells. Lastly, we showed that blocking NKp44 with a mAb enhanced NK cell-mediated lysis of the NK-insensitive AML cell line, THP-1.

This study points to the dual nature of the NKp44 receptor, previously considered as a classical NK cell activation receptor, [45] yet few studies have recognized this paradoxical functional response through NKp44. [40, 46] Paradoxical inhibition of human natural interferon-producing cells by the activating receptor NKp44 has been reported. [40] Furthermore, intratumoral NK cells, which were functionally impaired and associated with poor prognosis, displayed significantly higher expression levels of NKp44. [46] This dual character could be approached either from the side of the ligand expressed by target cancer cells or from the side of the NKp44 isoforms expressed by NK cells. Focusing on the level of the NKp44 isoforms expressed by the effector NK cells, we demonstrate for the first time that the “dual nature” of the NKp44 functional outcome could be explained by the expression of either NKp44^ITIM+^(NKp44-1) or NKp44^ITIM−^ (NKp44-2/NKp44-3) splice variants; i.e. on the effector side of the equation rather than the target side. Furthermore, we showed that this diverse NKp44 splice variant expression can influence the survival of AML patients. We therefore suggest that alternative splicing of NKp44 receptor mRNAs can regulate the immunosurveillance capacity and dictate the functional outcome of NK effector cell/target interactions in AML. A few recent studies have investigated the relationship between the expression of splice variants of innate immune cell receptors and outcome of cancer therapy. [37, 38] A previous study demonstrated that the NKp30/NCR3 splice variant profile can potentially be an effective tool to aid prognosis for GIST patients. [38] Therefore, investigating the expression of genes in cancer-associated immune cells (immunome) is imperative for designing personalized therapeutic approaches and predicting prognosis of patients.

On the level of the ligand, we and others published that PCNA is an inhibitory ligand for NKp44, and in this study, we further show that PCNA inhibits through the NKp44^ITIM+^ isoform but not the NKp44^ITIM−^ isoforms. [29, 31] The innate immune inhibition mediated by PCNA through interaction with NKp44-1 isoform directly correlates with the observation that cancer virulence is associated with PCNA overexpression by the cancer cells. Importantly, it is clear that the involvement of PCNA in cell cycle and proliferation is much more influential in promoting cancer virulence than its effect on immune suppression. [47, 48] Recently, a new activating cellular ligand of NKp44 was characterized as a specific isoform of MLL5. [30] This dichotomy between activating and inhibitory ligands of NKp44, which are expressed by target cancer cells, can lead to a diverse outcome of NK cell function. Since NKp44 also recognizes heparan sulfate proteoglycans as co-ligands, it was suggested that NKp44 could recognize cancer cell-produced damage-associated molecular patterns (DAMP) that can either activate (if containing MLL5) or inhibit (if containing PCNA and MHC class-I) NK cell function. [45] With regard to this theory, it is important to note that we showed the effect of NKp44-1 isoform on the NK insensitive HLA class-I^high^ THP-1 AML target cells, but we could not show it for the NK sensitive, HLA class-I^dull^ K562 CML target cells. Yet, the role of KIR receptors inhibiting through HLA class-I could not be excluded.

Recently, Siewiera et al. showed that IL-18 and TGF-β can influence NKp44 splice variant profiles and pointed to tissue specific editing of NKp44 in decidua basalis NK (dNK) cells, which are CD56^bright^. It is probable that tissue specific NK cells (CD56^bright^ and CD56^dim^) express different profiles of NKp44 splice variants, as well as other receptors, due to tissue-specific signals. On the other hand, the RNA expression profile of NKp44 splice variants 1 and 3 for unstimulated CD56^bright^ pNK and CD56^bright^ dNK cells is similar [49].

The use of *ex-vivo* expanded lymphokine-activated killer cells and NK cells for adoptive cancer immunotherapy has been studied since the 1980's. [50] In our hands, prolonged *ex-vivo* exposure of NK cells to IL-2/IL-15 preferentially induced the NKp44-1 splice variant and accordingly induced functional impairment of NK cells toward PCNA-expressing target cells. Recently, several studies described the use of *ex vivo* expanded NK cells for adoptive therapy of AML, [51] and most of these employed expansion with IL-2/IL-15. [52, 53] In view of our results, the use of IL-2/IL-15 should be considered carefully, since these cytokines induced dominant expression of the inhibitory NKp44-1 splice variant. It should be noted, that IL-12 was reported to reduce the levels of IL-2/IL-15-induced NKp44 expression, while inducing better cytotoxicity. [54],[55] Based on those results, the use of IL-12, NKp44-blocking mAb, or over-expression of NKp44-2/3 splice variants should be considered to potentially interfere with the suppressive effect attributed to the NKp44-1 variant.

To summarize, we show that a specific alternative splice variant of the NCR2 gene, namely the NKp44-1, is associated with poor survival of AML patients and that the balance between NKp44 isoforms NKp44-1 and NKp44-3 can lead to diverse function of NK cells and influence the outcome of the NK cell/target cell synapse formation. Taken together, our cumulative data indicate that an excess of NKp44-1 expression could result in functionally impaired NK cells in AML patients, and this expression profile may be an early prognostic indicator.

## MATERIALS AND METHODS

### TCGA samples

A total of 200 adult cases of spontaneously occurring, newly diagnosed AML from the LAML study (TCGA Data Portal; https://tcga-data.nci.nih.gov/tcga/) [56] were analyzed. RNA-Seq-V2 results were quantified through RNA-Seq by Expectation-Maximization (RSEM) using the “rsem.gene.normalized_results” and “rsem.isoforms.normalized_results” file types. All the data preprocessing and mentioned analyses were performed in the R statistical environment (http://www.r-project.org). [57] TCGA sample and liquate barcode: Project- Tissue source site- Study participant- Sample type/Vial- Portion/Analyte- Plate- Center (TCGA-AB-2842-03/A-01/T-0734-13). Sample type: 03 = Primary Blood Derived Cancer - Peripheral Blood. 09 = Primary Blood Derived Cancer - Bone Marrow. Analyte : T = total RNA, D = DNA. Percentage of NKp44 splice variants from total NKp44 mRNA was performed by adding the TCGA data of individual splice variants to total NKp44 expression and calculating the percentage of each. https://tcga-data.nci.nih.gov/datareports/codeTablesReport.htm?codeTable=sample%20type.

### PBMC and pNK cell isolation

PBMC isolation was performed with LSM® (MP Biomedicals, LLC). NK cell isolation kit (RosetteSep) was used to purify NK cells (purity>95%) from peripheral blood of healthy volunteer donors (recruited by informed consent as approved by the FCCC and BGU Institutional Review Boards). PBMC or NK cells were cultured in CellGro stem cell serum-free growth medium (CellGenix) supplemented as previously described [16] with 300 IU/ml human rIL-2 (Biological Industries) or 50ng/ml human rIL-15 (PeproTech). NK cell clonal expansion was performed as described. [58]

### Antibodies and reagents

PE anti-human-CD3 (clone:UCHT1) and PE anti-human-CD19 (HD37) were from IQproducts. Propidium Iodide (sc-3541, Santa Cruz), CFSE (Life Technologies), 7AAD, Brilliant-violet/Alexa-Fluor-647 anti-human-CD56 (HCD56), FITC anti-human-HLA-DR (L243), purified/APC/PE anti-human NKp44 (P44-8), and Pacific-Blue/PE anti-human CD16 (3G8) were from Biolegend. Alexa-Fluor-647 Goat anti-human IgG (109-605-088, Jackson) and NKp44-hFc and hFc were used at 60-100ug/ml according to their molecular weight as previously described. [16]

### Cell culture and cDNA constructs

We used the following cell lines: HeLa- derived from human cervical adenocarcinoma (ATCC CCL-2), K562- derived from human chronic myelogenous leukemia (CML, ATCC CCL-243), THP-1- derived from human acute myeloid leukemia (AML, ATCC TIB-202), NK-92 (ATCC CRL-2407) and KHYG-1- both derived from human NK cell leukemias. The NK-92 cell line was transduced with N-terminus FLAG-tagged NKp44 splice variants matching the following cDNA sequences: NKp44-1; NM_004828.3, NKp44-2; NM_001199509, NKp44-3; NM_001199510.1, using a retroviral transduction protocol as previously described. [29]

### RNA extraction, reverse transcription, real time PCR (qPCR) and primer set efficiencies

Total RNA was extracted using the RNeasy® Mini Kit (cat# 74104, Qiagen Ltd), and cDNA synthesis using 1μg of total RNA was performed as previously described. [36] qPCR analysis was performed using the TaqMan® Gene Expression Master Mix (cat# 4369016, Applied Biosystems). Each reaction contained 30ng of cDNA and was performed as previously described. [36] Expression levels of: total NKp44, NKp44-1, NKp44-2, and NKp44-3 (target genes) were normalized to GAPDH (reference gene PN4351370, Life Technologies). Calculation of gene expression was performed using the 2^−ΔCt^ method. The percentage of NKp44 splice variants from total NKp44 mRNA was performed by normalizing the expression of NKp44 splice variants to NKp44 total expression (reference gene), adding the 2^−ΔCt^ of each splice variant to a total NKp44 expression and calculating the percentage. Primer set efficiencies.

NKp44-total: Fw-primer: 5′-TGATGCTGGCTTCT TCACTG-′3, Probe: 5′-TCTGGTGGTATCTCCAGCCTC TGCCT-′3, Rev-primer: 5′-AGTCCAGGAGGT CTGTG TGG-′3, (eff%:98.6). NKp44-1: Fw-primer-1/3: 5′-ACCA TCCCTGTCC CTTCACAGCCAC -′3, Probe-1/2/3: 5′-GA CTCCTCGTAGCCAAGAGCCTGGTG-′3, Rev-primer-1: 5′-ACCATATGTCCCCCCACCAG-′3, (eff%:99.8). NK p44-2: Fw-primer-2: 5′-CTTCCTGTCCCTCTGCCT TC-′3, Probe NKp44-1/2/3: 5′-GACTCCTCGTAGCCAAGAGCCTGGTG-′3, Rev-primer-2/3: 5′-GATGC TGCAT GTGCCGATTCCTT-′3, (eff%:102.5). NKp44-3: Fw-primer-1/3: 5′-ACCAT CCCTGTCCCTTCACAGC CAC-′3, Probe-1/2/3: 5′-GACTCCTCGTAGCCAAGAG CCTGGT G-′3, Rev-primer-2/3: 5′-GATGCTGCATGTG CCGATTCCTT-′3, (eff%: 99.2).

### Cell stimulation, IFNγ assays and cytotoxicity assays

For IFNγ production assay, cells were stimulated in 96-well U-bottom plates (NUNC) pre-coated with 0.25 μg/mL anti-NKp44 mAb as previously described.^28^ Effector cells were mixed with target cells (1:3 ratio) and incubated for 18hr. IFNγ concentrations were assayed by standard ELISA assay (Biolegend). Cytotoxic activity of primary human NK cells was tested in a standard 5hr killing assay and calculated as previously described. [16] For NKp44 blocking experiments, NK cells were pre-incubated for 1h at 4°C with 20 μg/ml of anti-human NKp44 mAb (Clone P44-8) or with isotype control Ab.

### Immunocytofluorescence conjugation assay

Adherent target cells were harvested using 0.05% EDTA cell detachment solution to avoid ligand degradation by trypsin. Target cells were incubated on cell culture-treated 8-well μChamber slides (Ibidi) for 4-6 hours at 37°C, 5% CO2. Effector:target (2:1) cells were co-incubated for an additional 20 minutes. Cells were rinsed and then fixed using 1.6% v/v para-formaldehyde (Electron Microscopy Science). For F-actin labeling, samples were incubated in PBS solution containing 10 units/mL Alexa Flour 635-Phalloidin (Life Technologies), and 0.05% v/v Triton X-100. Nuclei were stained with 2mg/mL Hoechst 33342 (Sigma). [59]

### Statistical analysis

Graphics and statistical analysis were performed using GraphPad/Prism5 or Microsoft Office/Excel software. Percent survival vs “day of death” statistics were calculated using the Log-rank (Mantel-Cox) Test. mRNA expression statistics were performed using Unpaired t test, two-tail. *, p<0.05; **, p<0.01; ***, p<0.001. Pearson correlation, two-tail. *, p<0.05; **, p<0.01; ***, p<0.001.

## SUPPLEMENTARY FIGURES AND TABLES




