# Supplementary material for: Survival in acute myeloid leukemia is associated with NKp44 splice variants
Source: Oncotarget. 2016 Apr 18;7(22):32933–45. doi: 10.18632/oncotarget.8782 (PMC5078064; doi:10.18632/oncotarget.8782)
Supplement: Supplementary file 1 [file oncotarget-07-32933-s001.pdf]

Survival in acute myeloid leukemia is associated with NKp44 splice variants

SUPPLEMENTARY FIGURES AND TABLE

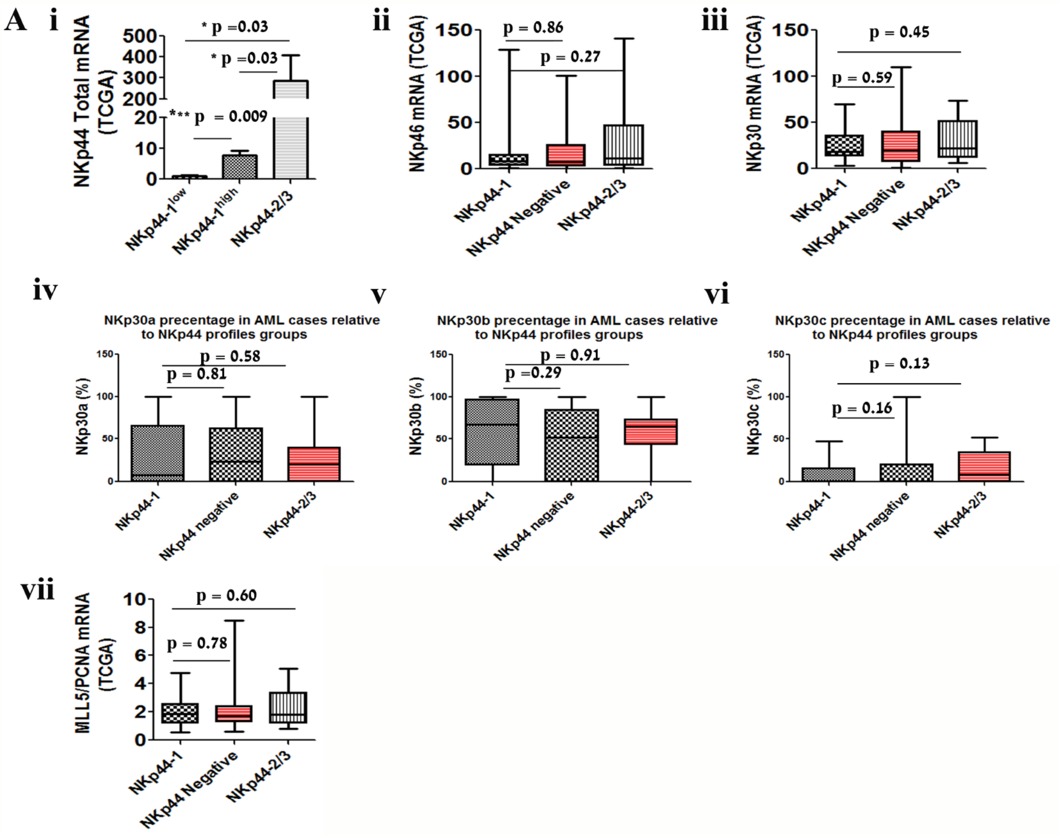

Supplementary Figure S1: gene expression of NCRs in NKp44 splice variants profiles groups: RNAseq analysis of PB samples obtained from AML patients (TCGA data). NKp46$^+$ cases with "day to death" information were grouped according to the NKp44 splice variant profile 1) NKp44-1 profile (n=24), 2) NKp44-2/3 profile (n=12) and NKp44 negative (n=60). **A.** NKp44 profile groups were analyzed for the mRNA expression levels of (i) NKp44, (ii) NKp46 and (iii) NKp30 and for each of the NKp30 splice variants percentage from total NKp30 (iv) NKp30a, (v) NKp30b and (vi) NKp30c (vii) MLL5/PCNA ratio. Differences of NKp46 NKp30, NKp30a, NKp30b, NKp30c and MLL5/PCNA ratio expression among the NKp44 profiles are not statistically significant. Unpaired t-test, two tail *, p<0.05; **, p<0.01; ***, p<0.001; Unpaired t-test, two-tail.

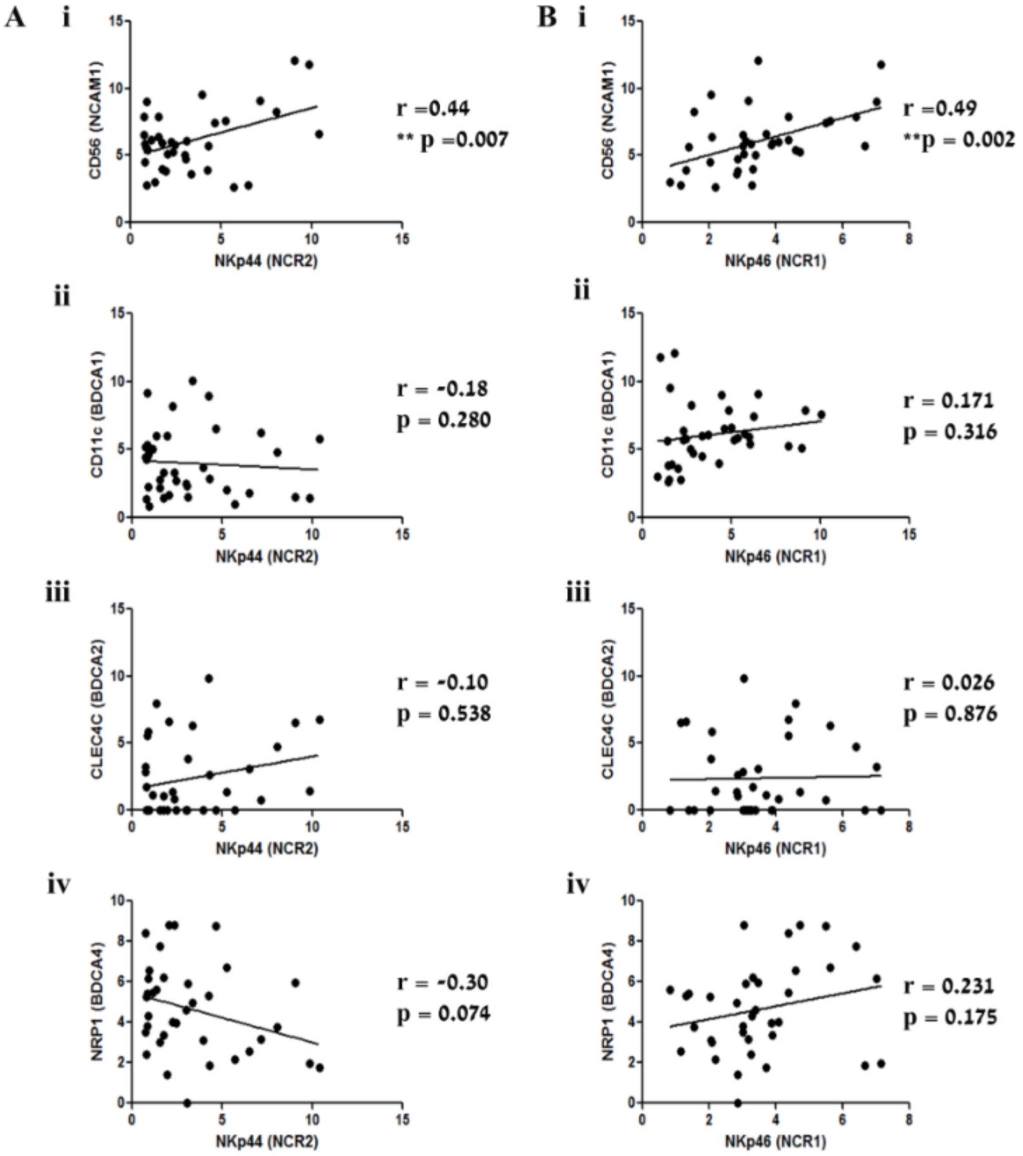

Supplementary Figure S2: Correlation analysis between the expression of NKp44 (NCR2) or NKp46 (NCR1) genes and defining cell marker genes in NKp46⁺NKp44⁺ AML cases. A. Correlation between NKp44 (NCR2) and (i) CD56 (NK cells). (ii) CD1C/BDC1 (Myeloid Dendritic cells), (iii) CLEC4/BDCA2 and (iv) NRP1/BDCA4 (plasmacytoid dendritic cells). **B.** Correlation between NKp46 (NCR1) and (i) CD56 (NK cells). (ii) CD1C/BDC1 (Myeloid Dendritic cells), (iii) CLEC4/BDCA2 and (iv) NRP1/BDCA4 (plasmacytoid dendritic cells). Pearson, two tail. N =36. * = $p < 0.05$, ** = $p < 0.01$.

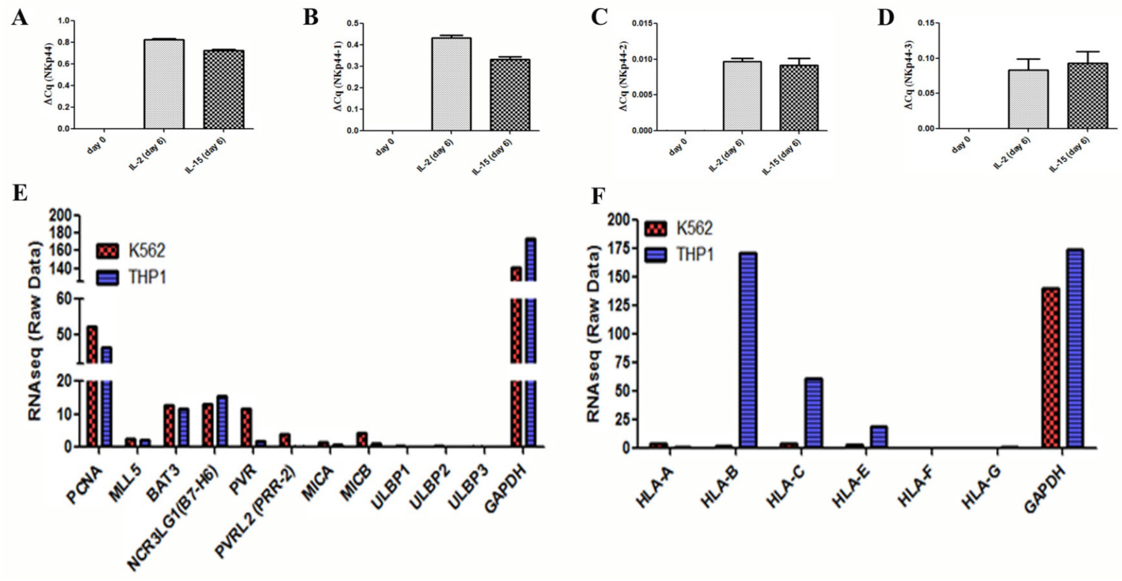

Supplementary Figure S3: qPCR analysis of NKp44 and NKp44 splice variants expression on day 0 and after 6 days of IL-2 or IL-15 culturing. A. NKp44, **B.** NKp44-1, **C.** NKp44-2, **D.** NKp44-3. RNAseq analysis in K562 and THP-1 cell lines: **E.** known cellular ligands of NK cells receptors (NKp44/NKp30/DNAM-1/NKG2D). **F.** Classical and non-classical HLA genes. Microarray datasets were obtained from GEO (http://www.ncbi.nlm.nih.gov/geo/). THP-1: GSM1125253, K562: GSM967005.

Supplementary Table S1: clinical data and known risk factors for AML in NKp46^{+} AML cases. NKp46^{+} AML Cases with “day to death” data were grouped according to the expression of NKp44/NCR2 by NKp44 profile, NKp44 positive (n-=36) and NKp44 negative (n=60) and NKp44 splice variants profiles NKp44-1(n=24) and NKp44-2/3 (n=12) AML cases. The clinical data was downloaded from the TCGA Data Portal (https://tcga-data.nci.nih.gov/tcga/

See Supplementary File 1
