# Supplementary material for: Survival in acute myeloid leukemia is associated with NKp44 splice variants
Source: Oncotarget. 2016 Apr 18;7(22):32933–45. doi: 10.18632/oncotarget.8782 (PMC5078064; doi:10.18632/oncotarget.8782)
Supplement: Supplementary file 2 [file oncotarget-07-32933-s002.docx]

**Supplementary – " Survival in Acute Myeloid Leukemia is associated with NKp44 variants"**


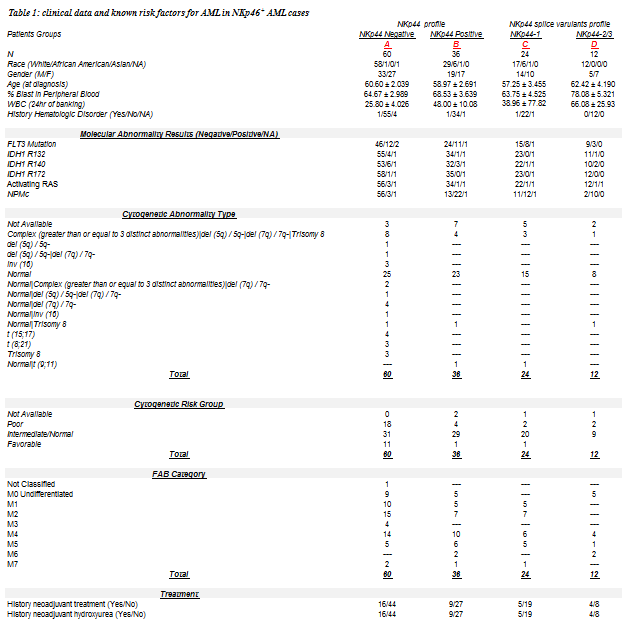


***Table 1: clinical data and known risk factors for AML in NKp46^+^ AML cases***. NKp46^+^ AML Cases with "day to death" data were grouped according to the expression of NKp44/NCR2 by NKp44 profile, NKp44 positive (n-=36) and NKp44 negative (n=60) and NKp44 splice variants profiles NKp44-1(n=24) and NKp44-2/3 (n=12) AML cases. The clinical data was downloaded from the TCGA Data Portal (<https://tcga-data.nci.nih.gov/tcga/>

* Gender: distribution of Male and Female was NOT significantly different for NKp44-1 vs NKp44-2/3 groups (p= 0.48) and for NKp44-1 vs NKp44-negative groups (p= 0.81). Fisher exact test.

* Age (at diagnosis): distribution of yes and no was NOT significantly different for NKp44-1 vs NKp44-2/3 groups (p= 0.37) and for NKp44-1 vs NKp44-negative groups (p= 0.39). Unpaired t-test, two tail.

* % Blast in Peripheral Blood: distribution of yes and no was NOT significantly different for NKp44-1 vs NKp44-2/3 groups (p= 0.06) and for NKp44-1 vs NKp44-negative groups (p= 0.86). Unpaired t-test, two tail.

* WBC (24hr of banking): distribution of yes and no was NOT significantly different for NKp44-1 vs NKp44-2/3 groups (p= 0.20) and for NKp44-1 vs NKp44-negative groups (p= 0.10). Unpaired t-test, two tail.

* History of hematologic disorders: distribution of yes and no was NOT significantly different for NKp44-1 vs NKp44-2/3 groups (p= 1) and for NKp44-1 vs NKp44-negative groups (p= 0.50). Fisher exact test.

* FLT3 Mutation: distribution of yes and no was NOT significantly different for NKp44-1 vs NKp44-2/3 groups (p= 0.7) and for NKp44-1 vs NKp44-negative groups (p= 0.25). Fisher exact test.

* IDH1 R132: distribution of yes and no was NOT significantly different for NKp44-1 vs NKp44-2/3 groups (p= 0.34) and for NKp44-1 vs NKp44-negative groups (p= 0.57). Fisher exact test.

* IDH1 R140: distribution of yes and no was NOT significantly different for NKp44-1 vs NKp44-2/3 groups (p= 0.26) and for NKp44-1 vs NKp44-negative groups (p= 0.66). Fisher exact test.

* IDH1 R172: distribution of yes and no was NOT significantly different for NKp44-1 vs NKp44-2/3 groups (p= 1) and for NKp44-1 vs NKp44-negative groups (p= 1). Fisher exact test.

* Activating RAS: distribution of yes and no was NOT significantly different for NKp44-1 vs NKp44-2/3 groups (p= 1) and for NKp44-1 vs NKp44-negative groups (p= 1). Fisher exact test.

* NPMc: distribution of yes and no was NOT significantly different for NKp44-1 vs NKp44-2/3 groups (p= 0.13); it was significantly different for NKp44-1 vs NKp44-negative groups (***p< 0.0001**)** with more "NPMc^+^" cases in the NKp44-1 group. We then assessed the NKp44 positive and NKp44-2/3 vs NKp44 negative groups. NKp44 positive ((***p< 0.0001 and NKp44-2/3 (***p< 0.0001) were significantly different then NKp44 negative group with higher incidence of "NPMc^+^" cases in NKp44 positive and NKp44-2/3 groups. This excludes the possibility that NPM1 mutation contributed to the low survival associated with NKp44-1 group**.** Fisher exact test.

* Cytogenetic risk group: distribution of intermediate/normal and poor was NOT significantly different for NKp44-1 vs NKp44-2/3 groups (p= 0.58); it was significantly different for NKp44-1 vs NKp44-negative groups (* p= 0.02) with more "poor" cases in the NKp44 negative group. Thus, it cannot contribute to the low survival associated with NKp44-1 group. Fisher exact test.
